# Supplementary material for: In Vitro Effect of Photodynamic Therapy with Different Lights and Combined or Uncombined with Chlorhexidine on Candida spp
Source: Pharmaceutics. 2021 Jul 30;13(8):1176. doi: 10.3390/pharmaceutics13081176 (PMC8398142; doi:10.3390/pharmaceutics13081176)
Supplement: Supplementary file 1 [file pharmaceutics-13-01176-s001.zip › pharmaceutics-1324877-SI.pdf]

pharmaceutics

MDPI

Supplementary Materials: In Vitro Effect of Photodynamic Therapy with Different Lights and Combined or Uncombined with Chlorhexidine on *Candida* spp.

Vanesa Pérez-Laguna, Yolanda Barrena-López, Yolanda Gilaberte and Antonio Rezusta

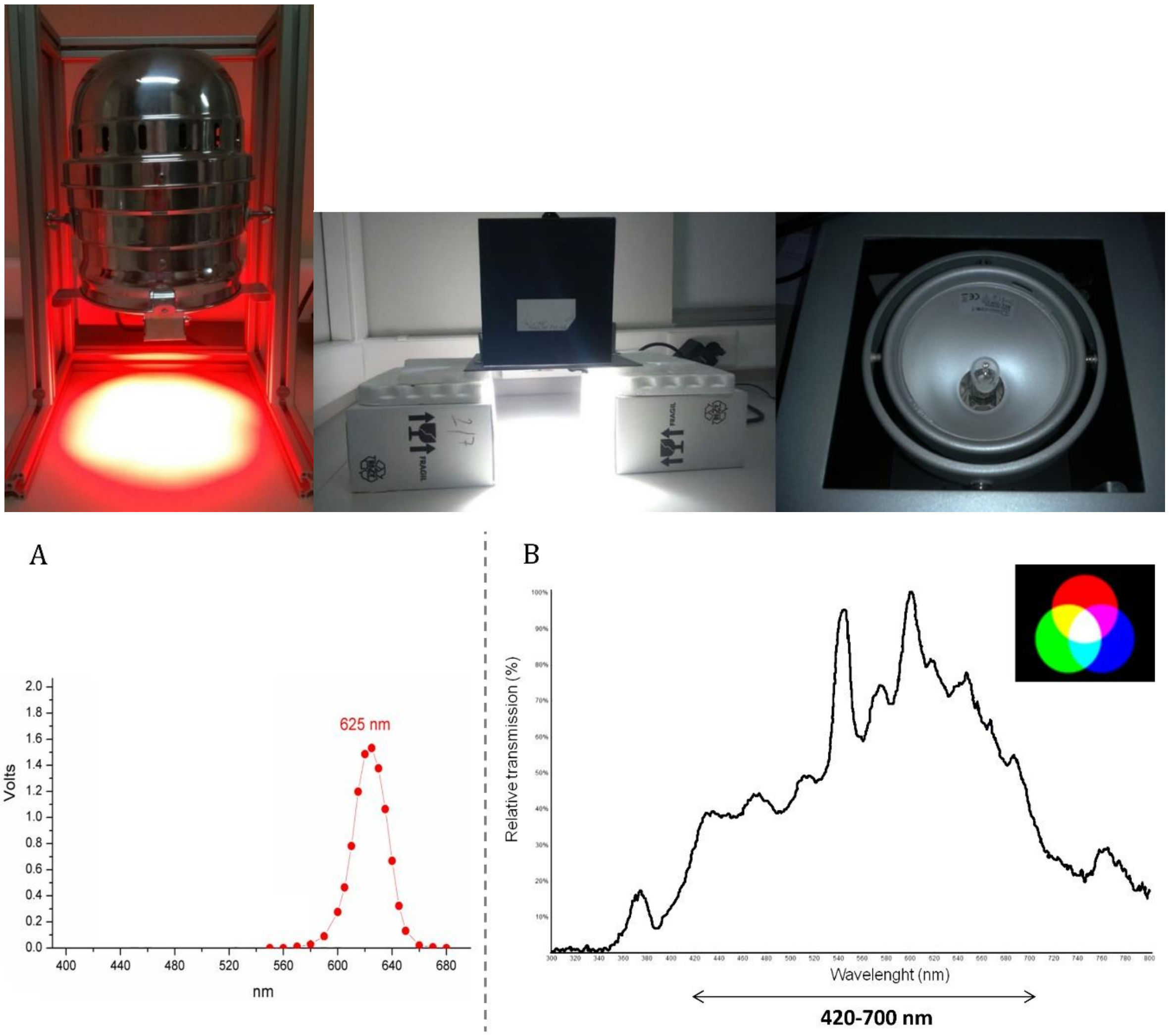

Figure S1. Photos and emission spectra of the lamps used in the photoinactivation experiments. **A**: graph of the emission spectrum of the red-LED lamp and **B**: emission spectrum of the white metal halide lamp.

I)

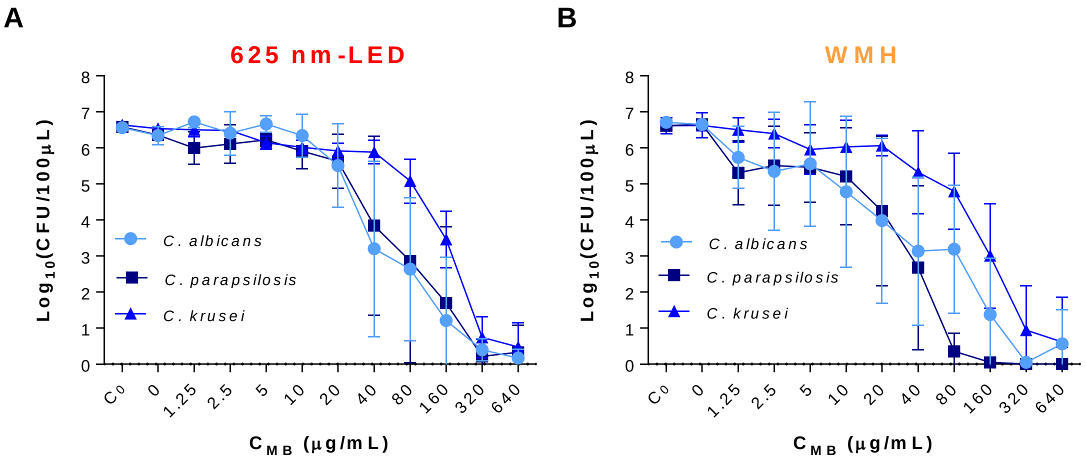
A
625 nm-LED
Log10(CFU/100µL)
C. albicans
C. parapsilosis
C. krusei
C0
0
1.25
2.5
5
10
20
40
80
160
320
640
CMB (µg/mL)
B
WMH
Log10(CFU/100µL)
C. albicans
C. parapsilosis
C. krusei
CMB (µg/mL)

II)

A

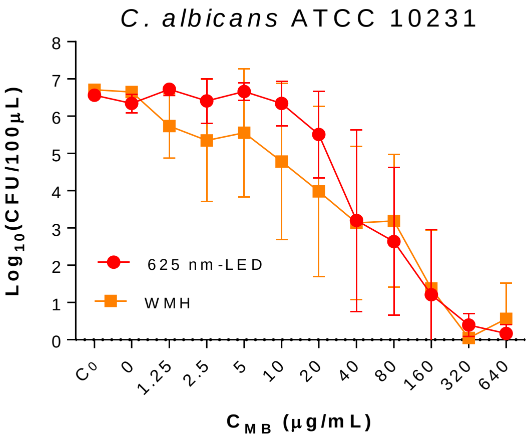

B

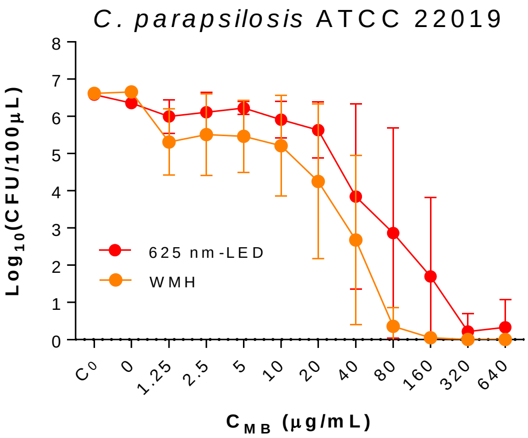

C

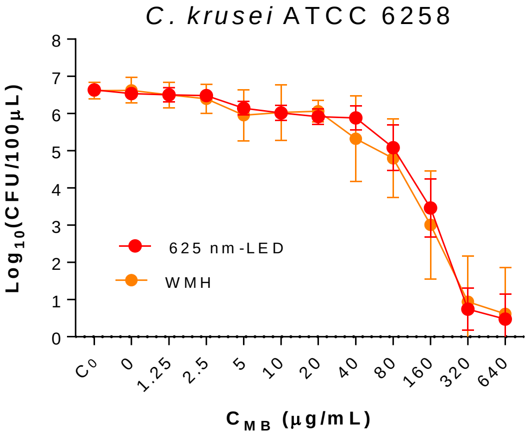

Figure S2. Photoinactivation of Candida spp. using MB-aPDT. **I**: Comparison of the response of yeast when they are irradiated with the red-LED lamp (left **A**) or with the WMH lamp (right **B**). **II**: Comparison of the response of each strain to irradiation with the two lamps (**A**: C. albicans; **B**: C. parapsilosis; **C**: C. krusei). The error bars represent the standard deviation calculated for five measurements. C0, initial inoculum control; LEDs, light-emitting diodes; MB, methylene blue; WMH, white metal halide.
